# Effect of Laughter Therapy on Depression and Quality of Life of the Elderly Living in Nursing Homes

**DOI:** 10.21315/mjms2020.27.4.11

**Published:** 2020-08-19

**Authors:** Mohammad Heidari, Mansureh Ghodusi Borujeni, Parvin Rezaei, Shokouh Kabirian Abyaneh, Korosh Heidari

**Affiliations:** 1 Community-Oriented Nursing Midwifery Research Center, Shahrekord University of Medical Sciences, Shahrekord, Iran; 2 Young Researchers and Elite Club, Abadeh Branch, Islamic Azad University, Abadeh, Iran; 3 Shariati Hospital, Tehran University of Medical Sciences, Tehran, Iran; 4 Treatment Affairs, Shahid Beheshti University of Medical Sciences, Tehran, Iran; 5 Treatment Affairs, Shahrekord University of Medical Sciences, Shahrekord, Iran

**Keywords:** laughter therapy, depression, elderly, quality of life

## Abstract

**Background:**

Mental disorders are common among the elderly with serious symptoms of depression and social isolation. This study was conducted to investigate the effect of laughter therapy (LT) on depression and quality of life (QOL) of the elderly living in Abadeh nursing homes.

**Methods:**

This is a controlled semi-experimental study with a pre-test, post-test design. Ninety eligible ones of the elderly living in the Abadeh nursing homes and from July to September 2017, entered the study. Some of the criteria for entering the study include being over 60 years old, orientation, not having blindness and deafness, lack of physical and mental problems. After determining the intervention and control groups, the scale of depression and QOL was administered to the subjects and their scores were collected in the pre-test.

**Results:**

Most of the study samples were in the intervention (35.55%) and control (37.77%) group in the age range of 60–69 years. In both intervention and control groups, respectively, 31.11% and 68.88% elderly were males and females. The mean scores of depression in the intervention group after LT (M = 2.57) were lower than those before the intervention (M = 6.87) [CI = −5.58–(–3.02)] and also the results of independent *t*-test showed a statistically significant difference before and after the intervention between the two groups (*P* < 0.001). The mean score of dimensions of QOL after LT was higher than that before in the intervention and there was a statistically significant difference in all dimensions with paired *t*-test (*P* < 0.001).

**Conclusion:**

Since the implementation of this programme could improve the mental status and QOL of the elderly, this method of therapy can be used as an alternative or complementary model to enhance the health of the elderly.

## Introduction

Old age is a sensitive period of human life in which the elderly are exposed to potential threats such as the increase in affliction with chronic diseases, loneliness and isolation, and the lack of social support, by which their individual independence is threatened in many cases regarding their physical and mental inabilities (1). In addition, as the population of the elderly grows, the prevalence of mental inabilities increased, and this important issue has affected the health and social care systems that are currently under tremendous pressures. The World Health Organization (WHO) estimated the total number of the elderly in the world to be around 700 million in 2006, which is expected to double in the next 40 years (2). Also in Iran, the population over 60 years of age will be about 10 million by 2020 and more than 26 million by 2050 (3). As nations aged, the problems of the elderly are continuously increasing, and depression, as the most common mental disorder and general problem, has made human life a major health problem for the elderly. Different difficulties and issues such as loneliness, loss of outdoor jobs, restructuring of the family system from the extended to the nuclear one, and rapid economic, social, and cultural changes have oriented the elderly to social isolation and consequently a diminished level of dignity and respect among families and the younger generation (4). Clinical researchers believe that depression often occurs in 12%–16% of the elderly, while 20%–30% of them sometimes show symptoms of depression (5). Depression lowers the quality of life (QOL) of the elderly and increases their dependence on others. In this regard, Ko and Hyun (6) reported that depression is a dangerous disease because they believe it increases the risk of death and disease-related deaths and reduces the QOL. Nurses play a very important role in seeking out the symptoms of depression in the elderly and providing them with nursing interventions and can enhance their QOL. With increasing age, there will be changes in various aspects such as physical weakness, impaired brain function, mental disorders such as loneliness, chronic illnesses and so on. Therefore, due to the above problems, they are susceptible to injury and decrease in their QOL, which this necessitates that they be paid attention and receive effective care systems in preventing unpleasant events (7). QOL, which is a new indicator of the provision of health, medical and care services to different population groups, especially the elderly, is a kind of individual feeling of well-being and comes from the satisfaction or dissatisfaction with different aspects of life that are important to the individual. This concept encompasses the physical, psychological, social and spiritual functioning of individuals and is dependent on their political, cultural, economic and spiritual beliefs (8). QOL in old age is more important due to the specific physiological conditions of the elderly. QOL assessment has been a useful way of identifying the health and well-being of the elderly as well as a criterion for determining the impact of provided treatments and cares (9). Laughter therapy (LT) has a special place in alternative and complementary medicine, in such a way that laughter is a universal, effective and low-cost drug with no side effects. The therapeutic benefits of laughter include improved blood circulation, skeletal-muscular, gastrointestinal, and respiratory systems of the body, as well as hormones regulation, sleep and rest cycle regulation, and enhanced immune system performance (10). By reducing stress hormones like cortisol and increasing body readiness for coping with different kinds of distress, laughter can probably remove symptoms of disease like fatigue and consequently improve QOL (11). Taking care of the needs of the elderly and utilising their talents and abilities are key elements to bringing joyousness and happiness to the old age. Since pleasant emotions are very important to people’s health and QOL, having a level of laughter can dramatically affect their health and social relationships with others. Researchers believe that if the elderly are regularly made to laugh, their sociability can be reinforced (12). The use of LT and physical activities in any age conditions can bring forth biological changes to enhance physical and mental health and increase self-esteem (10). Therefore, in view of the prevalence of depression and QOL problems in the elderly and the noticeable importance of LT in their health, this study investigated the effect of this method on the level of depression and QOL of the elderly.

## Methods

### Study Design

**Figure 1:**
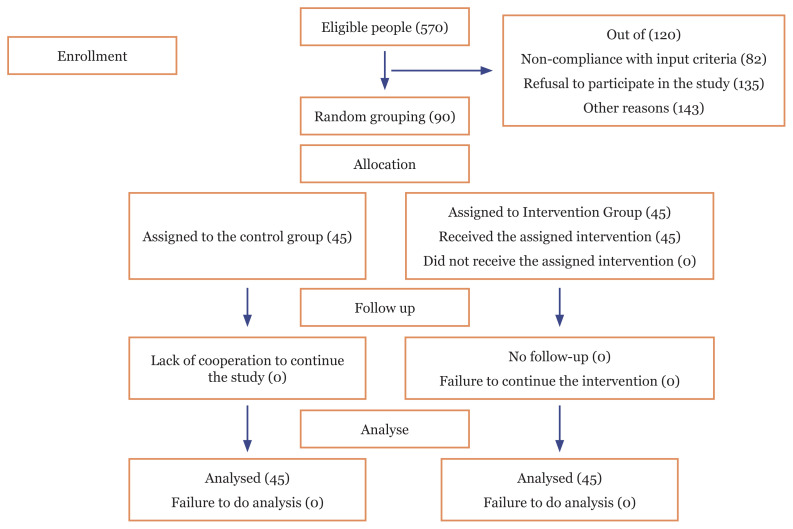
Sampling process of the elderly living in nursing homes

This is a controlled semi-experimental study with pre-test, post-test design in 2017. The study population consisted of the elderly living in Abadeh 24 h nursing homes. Considering the 95% confidence level and 80% test power as well as a maximum of 45-unit joint variance between the intervention and control groups, the sample size was taken to consist of 60 persons; it was considered of 90 persons with the possibility of some subjects’ withdrawal that from July–September 2017, they entered the study. At first, using a census out of 1200 subjects, 570 samples were considered eligible and then using random sampling, 90 persons were selected and randomly divided into control and intervention groups, so that 45 persons through simple random allocation were assigned in each group (Figure 1). In the above study, coin throwing method was used for simple randomisation. It is commonly used to generate random sequences in the two-group trials in such a way that one of the study groups considers the milk and the other group as the line and based on the size of the sample, the same number of stocks are scattered and they will randomly into two groups. Geier et al. (13) also in their study used the coin toss to produce a random sequence. In order to hide accidental allocation, non-transparent envelopes are stamped and sealed with the random sequence was used. After creating random sequences with the method based on the sample size, number of non-transparent envelope provided and each randomly generated sequences were recorded on a card and the card was placed inside the envelopes respectively. In order to maintain the random sequence, numbered pockets on the exterior was the same way. Finally, the lids of the letter envelopes were glued and placed inside a box, respectively. At the beginning of the registration of the participants, according to the order of entry of the participants who are eligible to study, one of the mentioned chapters will be opened in order. An example of this approach can be found in the Smilde et al. (14). In order to implement the random allocation process, a person other than the main researchers was used to create the random programme to reduce the possible fluctuations. Blinding methods were used to analyse the information so that the analyst did not know which test group and which control group.

After determining the intervention and control groups, depression and QOL scale was administered to the subjects in both groups and the subjects’ scores were collected in the pre-test. Then, 10 sessions of LT were administered to the intervention group subjects 3 times a week. Each session lasted 1 h. Intervention in each session was performed in modes of playing musical and visual slides and humorous video clips (30 min), as well as holding happy and joyous games with prizes of humour telling (15 min) and joke telling (15 min). Patients had active and interactive participation in meetings by taking part in competitions and telling jokes. There were no plans for the control group, but to regard the ethics and motivate cooperation in completing the questionnaires, they were promised that such sessions will be implemented for in the future as well. After the training sessions were ended, the researcher gathered the subjects of both groups in one session and completed the questionnaires for them again. The criteria to enter the study included being over 60 years of age, having complete knowledge of time, place, and person, having no blindness and hearing loss, having no history of psychiatric hospitalisation and psychiatric treatment, having no experience of grief for the past six months, staying in a sanatorium for at least 6 months, not being afflicted with acute illnesses, and not taking temporary leave for more than a week during the study period.

### Data Collection

Data were collected using two questionnaires of the elderly’s depression and SF-36 QOL. It should be noted that the first part of the questionnaires contained demographic questions. The SF-36 QOL questionnaire was first designed by Ware and Sherbourne in the United States in 1992 and also its reliability and validity were confirmed (15, 16). The questionnaire included 36 questions on eight subscales of physical functioning, limitations on role playing due to physical problems (physical role playing), limitations on role playing due to emotional problems (emotional role playing), social functioning, mental health, vitality, physical pain and general health (general understanding of health). Each of these eight subscales scores from 0 to 100, with higher scores indicating better QOL. Three-choice questions with scores (of 0, 50 and 100), five-choice questions with scores (of 0, 25, 50, 75 and 100) and six-choice questions with scores (of 0, 20, 40, 60, 80 and 100) were intended. The mean score of QOL subscales was 50, with higher and lower scores indicating high and low QOL, respectively (16, 17). The questionnaire was first translated and standardised in Persian by Montazeri and colleagues in Iran, with a variable Cronbach’s alpha value of 0.77 to 0.90 for different dimensions of the instrument (15), and also the validity and reliability of this scale have been repeatedly demonstrated by other Iranian researchers (15, 18). Besides, the Cronbach’s alpha value of the above-mentioned scale for the present study was 0.81. The questionnaire for depression assessment consists of 15 questions from the elderly’s standardised depression scale designed and validated for the assessment of depression in the elderly (19, 20). In this questionnaire, a score of 0–4 indicates no depression, 5–8 mild depression, 9–11 moderate depression and 12–15 severe depression. According to the study of Malakouti et al. (20), the instrument had a Cronbach’s alpha value of 0.9 and also its validity was 0.9 by factor analysis. The Cronbach’s alpha value of this instrument was 0.88 in the present study, indicating that the instrument has good reliability.

### Data Analysis

Data were analysed by SPSS/17 using descriptive statistics (frequency distribution table) and inferential statistical tests independent *t*-test, ANOVA, Chi-square and Pearson correlation coefficient. The assumptions raised in the study and the research questions that all of which were evaluated are as follows: i) The severity of depression in the intervention group after LT is less than the control group; ii) LT has an effect on reducing the average score of depression; iii) LT increases the QOL in the intervention group; iv) What is the relationship between the QOL of the elderly in both groups and demographic characteristics?; v) What is the relationship between the depression of the elderly in both groups with the demographic characteristics?; and vi) What is the relationship between QOL and depression in both groups?

## Results

The results indicated that the majority of the study samples were in the intervention (35.55%) and control (37.77%) groups in the age range of 60–69 years. In this study, in both control and intervention groups, 31.11% of the study participants were male and 68.88% were female. Other individual characteristics of the units under study are listed in Table 1. The results of Table 2 show that in the intervention group, the majority of the elderly (40%) had mild depression before LT, but they had normal depression scores and showed reduced depression symptoms after the intervention (66.66%). The results of Table 3 show that the mean scores of depression in the intervention group after LT were lower than those before the intervention and also the results of independent *t*-test showed a statistically significant difference before and after the intervention between the control and intervention groups (*P* < 0.001). The mean score of dimensions of QOL after LT was higher than that before in the intervention and there was a statistically significant difference in all dimensions with paired *t*-test (*P* < 0.001). The total mean score of QOL dimensions for the intervention group was 47.15 (16.02) before the intervention, which increased to 59.96 (17.58) after the intervention and the paired *t*-test showed a statistically significant difference (*P* < 0.001) (Table 4). There was a significant and inverse relationship between QOL and depression in the control (*P* < 0.001 and *r* = −0.712) and intervention (*P* < 0.001 and *r* = −0.76) groups. More precisely, QOL decreases with increasing depression. Pearson correlation coefficient showed that there was a direct and statistically significant relationship between QOL and sex, marital status, education level and economic status in both control and intervention groups (*P* < 0.001). The results of the Chi-square test and one-way ANOVA test also showed that there was a statistically significant difference between depression and sex, marital status, education level and economic status in both groups (*P* < 0.001).

## Discussion

The purpose of this study was to determine the effect of LT on depression and QOL in the elderly living in sanatoriums. In this regard, it was found that LT had a significant role in reducing depression and enhancing QOL. LT is one of the most important methods of adjustment, reduction of depression and improvement of QOL (21). The results of this study showed that before LT, the majority of the subjects in the intervention group (40%) had mild depression, which later most of the elderly had no symptoms of depression. Generally, laughter enhances physical, mental, emotional and mental health and frees one from mental stresses (22). The results of this study are in line with research on the effect of LT on psychological variables in the elderly population. According to the results of a cross-sectional study conducted in Qom, the rate of depression was 48.3% (23). In their study of the effect of LT on life expectancy in the elderly in Tehran, Madadi et al. (24) confirmed that LT had an impact on their life expectancy and depression. In another study, Khoushkonesh and Keshavarz (25) made emphasis on the effectiveness of happiness education on reducing anxiety and improving cognitive symptoms of depression like life expectancy and the relationship between happiness and mental health components in the elderly. In the study of Chiang et al. (26), the effect of memory telling and laughter was noted on the reduction of depression and feeling of loneliness. In addition, Shahidi et al. (27) conducted a study entitled ‘The effectiveness of LT on the enhancement of general health of the elderly’, whose results indicated that LT increased the level of general health and decreased the old men’s depression (27). The results of a study by Moshtagh et al. (28) in multiple sclerosis patients showed that LT reduced the severity of fatigue and depression in multiple sclerosis patients and also indicated that the mean score of depression severity decreased after LT. Besides, the results of studies of Chodzko-Zajko et al. (29) and Tseng et al. (30) showed that physical activity affects mood and motor and cognitive functioning. If aging is to be a positive event, a longer life should be accompanied by constant opportunities for health, participation and safety. Dynamic aging is the process of optimising opportunities for health, participation and security to enhance the QOL of the elderly. For dynamic health, QOL is an integral component (31). In the present study, the mean score of QOL dimensions after LT for the intervention group increased compared to the one before that. In their study on the patients’ population, Rad et al. (32) showed that patients in the LT group had a better QOL than those in the control group during the treatment. There was a significant and inverse relationship between the QOL and depression in the control group (*P* < 0.001 and *r* = −0.712) and intervention one (*P* < 0.001 and *r* = −0.76). More accurately, with increasing depression, QOL decreases. This finding is consistent with other studies including the study by Bazrafshan et al. (33) and Fleming et al. (34). For example, the study conducted by Bazrafshan et al. (33) to determine the QOL in the elderly in Shiraz found that there was a significant relationship between different dimensions of QOL and depression (33, 34). The mental dimension of the elderly’s life seems to play an important role in their QOL. Changes occurring in middle age also cause mental disorders (35). Pearson correlation coefficient revealed that there was a direct and statistically significant relationship between QOL and sex, marital status, education level, and economic status in the intervention and control groups. In this study, the mean score of overall QOL of men was higher than that of women. One reason may be that due to many child births, women may have various diseases such as osteoporosis when they become old, which can have a negative effect on their QOL. Other causes include the limited physical activity of women outside the home (before entering the sanatorium) and traditional beliefs that recognise women as caregivers for children and husbands, which can show differences in the QOL of men and women. Also, women’s more sensitivity to coping with adverse events and their menopausal period have been known to be effective in this regard. In the study of Min et al. (36) and Tajvar et al. (37), men had a better QOL than women. The isolated elderly had higher mean scores than the others (the single, married and deceased-spouse ones) because the isolated ones had accepted the accommodation in the sanatorium as they wished, and because the rest of them (the single, married and deceasedspouse ones) did not enter the sanatorium voluntarily, the QOL of the isolated was higher than that of the others. The mean score of QOL for the illiterate elderly was lower than that of the others (under high school diploma, diploma, and academic degree), which may be due to limiting them in sanatoriums because most of them have been transferred to the sanatoriums thanks to their families and relatives’ unwillingness to look after them, which affects them mentally and reduces significantly their QOL compared to the other groups. Besides, the mean score of QOL of the elderly with academic degrees was lower than that of the elderly with under high school diploma and diploma, which may be due to their lack of enjoying more facilities and factors affecting their QOL. The study by Ahangari et al. (38) showed that as education increases, QOL increases as well. In addition, according to the research findings, economically independent people had a higher QOL than the dependent ones, which was confirmed in the study of Habibi et al. (39) as well. In this study, depression was less prevalent in the female elderly, with numerous studies showing various results in this regard (40, 41). The less prevalence of depression in the Iranian female elderly may be attributed to the continuation of family life-related activities, but, getting old gradually, the majority of men have been retired and gotten away from social activities and their colleagues, leading to a sense of inefficiency, loneliness, and inability, especially for those who consider their self-esteem and dignity dependent on work, which develops depression in them accordingly. The results of this study indicate that there is a higher prevalence of depression in the elderly with a high school diploma and lower compared to the ones with academic degrees. The results of the Alizadeh-Khoei’s (42) study on the Iranian elderly living in Australia also confirm this result. This result seems somewhat acceptable, since literate people are often able to access more information and information resources in any field, which affects their learning and improving skills or changing behaviours. The results of this study indicate a higher prevalence of depression in the elderly who were living alone due to the death of their spouse, divorce, or separation, which is consistent with other studies (40, 43). The results of the above studies indicate the impact of living alone on the level of depression in the elderly. The economic situation is effective on the elderly’s level of depression, as in the current study, the highest rate of depression was observed in those with poor economic status.

### Limitations

One of the limitations of this study is the use of self-reporting tools, based on which the subjects may not be completely honest in expressing their problems and responding to questionnaires. Also, considering the point the present study is based on the elderly and that they have very different cultural, social and family backgrounds, it does place limitations on the generalisation of findings, interpretations, and aetiologies of the variables under study, which need to be taken into consideration. It is therefore suggested that future research be done on the elderly with more homogenous cultural, social and family settings.

## Conclusion

With the increase in the population of the susceptible and vulnerable elderly group, as well as due to the importance of improving life and health in these individuals, health professionals should consider strategies to improve their QOL and reduce their psychological problems. According to the results of this study, a period of LT is one of the low-cost, safe, and non-invasive interventions that decrease the depression of the elderly by increasing endorphin and improving mood. Therefore, it is necessary that this treatment program be used to improve the QOL of the elderly.

## Figures and Tables

**Table 1:** Sample characteristics

Variable		Control group		Case group	
	
		*n*	%	*n*	%
Age	60–69	17	37.7	16	35.5
	70–79	13	28.8	14	31.1
	80–89	10	22.2	10	22.2
	90–99	5	11.1	5	11.1
Gender	Female	31	68.8	31	68.8
	Male	14	31.1	14	31.1
Marital status	Single	2	4.4	3	6.6
	Married	5	11.1	8	17.7
	Divorced	6	13.3	8	17.7
	Widow	32	71.1	27	60
Level of education	Illiterate	25	55.5	12	26.6
	Less than college	14	31.1	12	26.6
	College or higher	6	13.3	5	11.1
Economic status	Poor	14	31.1	18	40
	Medium	14	40	25	55.5
	Good	13	28.8	2	4.4
Satisfaction with nursing home	Yes	12	26.6	15	33.3
	No	10	22.2	14	31.1
	Somewhat	23	51.1	16	35.5
Satisfaction with family members	Yes	9	20	12	26.6
	No	22	48.8	18	40
	Somewhat	13	28.8	15	33.3
Previous job status	Employee	15	33.3	14	31.1
	Housewife	25	55.5	26	57.7
	Worker	4	8.8	2	4.4
	Self-employment	0	0	2	4.4
	Farmer	1	2.2	0	0
	Unemployed	0	0	1	2.2
Duration of stay in a nursing home	Less than 1 year	7	15.5	8	17.7
	1–5 years	15	33.3	13	28.8
	More than 5 years	23	51.1	24	53.3

**Table 2:** Frequency of depression level before and after LT

Level of depression	Control group				Case group			
	
	Before intervention		After intervention		Before intervention		After intervention	
			
	*n*	%	*n*	%	*n*	%	*n*	%
Normal	21	46.6	20	44.4	13	28.8	30	66.6
Mild	14	31.1	15	33.3	18	40	11	24.4
Moderate	6	13.3	4	8.8	5	11.1	1	2.2
Major	4	8.8	6	13.3	9	20	3	6.6

**Table 3:** Comparison of mean depression scores before LT

Group	Control				Case				Statistical test	

									Independent *t*-test	*P*
Depression	M (SD)	*n*	95% CI for the mean difference		M (SD)	*n*	95% CI for the mean difference			
				
Stages			Upper bound	Lower bound			Upper bound	Lower bound	1.53 df = 44	0.231
		
Before intervention	5.7 (3.57)	45	1.86	−1.22	6.87 (3.62)	45	−3.02	−5.58		
		
After intervention	6.02 (3.78)	45			2.57 (2.35)	45			−5.22 df = 44	< 0.001

**Table 4:** Comparison of elderly QOL dimensions

Dimensions of QOL-	Before intervention	After intervention	CI 95%for the mean difference		Statistical test		
			
	M (SD)	M (SD)	Upper bound	Lower bound	*P*	Independent *t*-test	df
General health	35.81 (17.60)	42.20 (17.82)	13.93	1.15	> 0.001	1.69	44
Physical performance	46.54 (25.62)	56.42 (30.42)	21.86	2.1	> 0.001	1.64	44
Playing the physical role	51.32 (17.9)	65.23 (40.41)	27.23	0.59	> 0.001	2.08	44
Playing an emotional role	51.17 (17.1)	61.25 (16.65)	17.26	2.9	> 0.001	2.80	44
Social performance	58.49 (18.97)	69.54 (53.14)	28.05	5.95	> 0.001	1.3	44
Physical pain	52.36 (19.91)	45.41 (20.7)	1.69	15.59	> 0.001		44
Vital power and energy	49.98 (13.66)	52.61 (10.51)	7.81	2.55	> 0.001	1.01	44
General understanding of health	49.14 (17.21)	63.65 (42.63)	28.35	0.67	> 0.001	2.09	44
Overall mean dimensions of QOL	47.15 (16.02)	59.96 (17.58)	19.97	5.65	> 0.001	3.57	44
